# Application of Electrospray in Preparing Solid Lipid Nanoparticles and Optimization of Nanoparticles Using Artificial Neural Networks

**Published:** 2020

**Authors:** Elaheh Shanaghi, Mahdi Aghajani, Fariba Esmaeli, Mohammad Ali Faramarzi, Hoda Jahandar, Amir Amani

**Affiliations:** 1. Department of Pharmaceutical Biotechnology, Faculty of Pharmacy, Tehran University of Medical Sciences, Tehran, Iran; 2. Department of Biotechnology, Faculty of Advanced Science and Technology, Tehran Medical Sciences, Islamic Azad University, Tehran, Iran; 3. Department of Medical Nanotechnology, Faculty of Advanced Technologies in Medicine, Tehran University of Medical Sciences, Tehran, Iran; 4. Pharmaceutical Sciences Research Center, Tehran Medical Sciences, Islamic Azad University, Tehran, Iran; 5. Natural Products and Medicinal Plants Research Center, North Khorasan University of Medical Sciences, Bojnurd, Iran; 6. Medical Biomaterials Research Center (MBRC), Tehran University of Medical Sciences, Tehran, Iran

**Keywords:** Nanoparticles, Neural networks, Particle size, Polymers

## Abstract

**Background:**

Electrospray (Electrohydrodynamic atomization) has been introduced as a novel approach to prepare nanoparticles. This work aimed to prepare SLNs through electrospray and evaluate factors affecting particle size of prepared Solid Lipid Nanoparticles (SLNs).

**Methods:**

SLNs were prepared by electrospray method. To study the factors affecting particle size of SLNs, Artificial Neural Networks (ANNs) were employed. Four input variables, namely, Tween 80 concentration, lipid concentration, flow rate, and polymer to lipid ratio were analyzed through ANNs and particle size was the output.

**Results:**

The analyzed model presented concentration of Tween 80 (surfactant) and lipid as effective parameters on particle size. By increasing surfactant and decreasing lipid concentration, minimum size could be obtained, while flow rate and polymer to lipid ratio appeared not to be effective.

**Conclusion:**

Concentration of surfactant/lipid plays the most important role in determining the size.

## Introduction

The most popular method to prepare Solid Lipid Nanoparticles (SLNs) is using high pressure homogenization. However, this method has considerable limitations, such as potential damage to sensitive molecules and low encapsulation efficiency. This has provided the rationale to look for alternative methods ^1^. Electrospray is a method of converting liquids into small particles, using an electric field. Desired particle size and shape can be achieved by optimization of variables like liquid flow rate and applied voltage. Electrospray represents unique features such as being a simple and single-step method with the ability to control many processing parameters. Also, adverse effects on bio molecules which is commonly observed when using other methods have been resolved with electrospray^2^.

There is very limited number of papers about preparation of lipid-based nanoparticles using electrospray. SLNs from stearic acid and ethyl cellulose to carry tamoxifen were prepared with particle size <1 *μm*. This research demonstrated entrapment efficiency of about 70% and zeta potential of −29.1±1.3 *mV* ^3^. Also, preparation of SLNs by electrospray with the purpose of flavor encapsulation led to a size <100 *nm* and entrapment efficiency of 69.5% ^4^. The present paper aimed to investigate the important parameters affecting particle size of SLNs prepared through electrospray. Artificial Neural Networks (ANNs) were utilized for this purpose. Four parameters (*i.e*. Tween 80 concentration, lipid concentration, flow rate, and polymer to lipid ratio) were considered as the inputs and the particle size was considered as the output.

## Materials and Methods

### Materials

Ethyl Cellulose (EC) was obtained from Sigma-Aldrich (USA). Octadecylamine, Tween 80, and chloroform were purchased from Merck chemicals (Germany).

### Preparation and characterization of Nanoparticles

EC and octadecylamine were dissolved in chloroform (Table 1). Glass syringe with a metal tip was used as the nozzle. The solution was fed through 16 *cm* PTFA tube which was connected to a needle gauge 26. A high voltage (around 7 *kV*) was applied between the metal needle tip and a copper ring electrode. The lipid particles were collected in a dish of 18 *cm* below the needle tip which contained 5 *ml* of distilled water and Tween 80.

**Table 1.** The training parameters used with INForm v4.02

No. of nodes in hidden layer		2
Backpropagation type		Incremental
Transfer function		
	Output	Asymmetric sigmoid
	Hidden layer	Asymmetric sigmoid

Dynamic Light Scattering (DLS) was employed in measuring particles size distribution after the preparation of particles using Scateroscope I (K-ONE, Korea).

### Artificial neural networks modeling

Optimization of the particles was carried out using a commercially available software package (INForm v4. 02, Intelligensys, UK). The results of the ANNs model are represented as three-dimensional graphs (*i.e*. response surfaces) that show effect of two inputs on the output. The current study consisted of four independent input parameters [namely, flow rate (*ml/h*), Tween 80 concentration (%), polymer to lipid ratio and lipid concentration (%)], and one output parameter (Particle size *nm*).

First, 23 samples with random values for each of the parameters were prepared, of which 17 preparations were used as training data set to train the ANNs for finding the relationships between input and output variables. Also, two data sets, chosen randomly by the software, were applied as test data for prevention of overtraining during the learning process (*i.e*., 10% of training data sets). Training parameters applied for modeling particle size are shown in table 1, and details of the parameters have been described previously ^5^. Also, to evaluate the predictive ability of the created model by ANNs, six remaining data sets were used as an unseen (Validation) data set (Table 2). A model with better predictability shows the correlation coefficient (R^2^) near one for the unseen data set.

**Table 2.** The unseen data sets utilized in ANNs modeling

**Flow rate (*ml/h*)**	**Lipid concentration (%)**	**Tween 80 concentration (%)**	**Polymer to lipid ratio**	**Observed particle**	**Predicted size (*nm*)**
**1.0**	0.6	0.5	0.3	3860	3799
**1.0**	0.2	1.0	0.2	10	456
**0.8**	0.3	1.0	0.2	246	459
**0.8**	1.0	0.5	0.2	3560	5444
**1.0**	0.5	0.5	0.5	5280	4432

## Results

The best predictive ANNs model presented R^2^ values of 0.73, 0.95 and 0.94 for training, test, and validation (unseen) data sets, respectively. Response surfaces, illustrated as 3D plots, were used to reveal information about impact of two input parameters on the particle size when values of the two other input parameters were fixed at a mid-level value.

**Figure 1.**
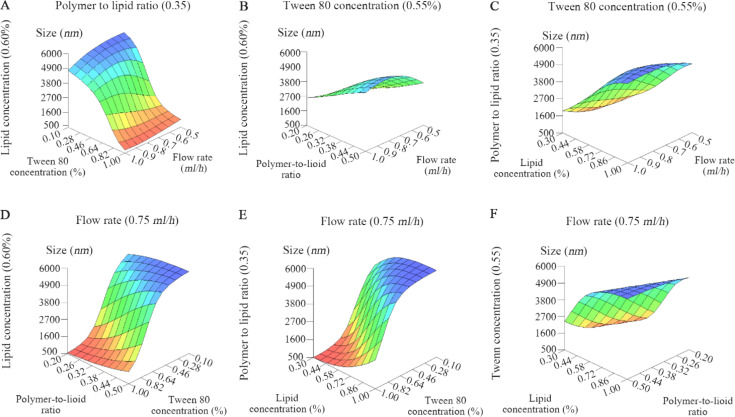
3D plots of the particle size predicted by the ANNs model fixed at mid-range value of two independent parameters.

As observed in figure 1A, two input parameters (Polymer to lipid ratio and lipid concentration) were fixed at medium values to study the effect of Tween 80 concentration and flow rate on the particle size. Figure 1 indicates that the particle size decreases dramatically when amount of Tween 80 concentration increases, while flow rate has no important effect on the particle size. Figure 1B presents how polymer to lipid ratio and flow rate have influenced the particle size while the two other inputs were fixed. From the figure, although flow rate and polymer to lipid ratio cause some fluctuations in size, the overall impact on the particle size is minimal. As illustrated in figure 1C, lipid concentration can affect particle size in a way that a gradual increase in particle size was detected by increasing lipid concentration. The figure also indicates that the effect of flow rate on particle size is negligible, as reported above. Figure 1D exhibits the effects of Tween 80 concentration and polymer to lipid ratio on particle size. From the details, particle size drops very slightly when ratio of polymer to lipid decreases. The figure also highlights the important role of Tween 80 concentration on particle size. Evaluation of Tween 80 and lipid concentration (Figure 1E) shows that both input parameters noticeably affect particle size. While increasing Tween 80 leads to decrease in size, increasing lipid concentration can form larger particles. The plots of figure 1F elucidate that the polymer to lipid ratio did not affect particle size. However, lipid concentration has an important role in particle size.

The most remarkable result emerged from all the plots is that from the four parameters studied, Tween 80 and lipid concentration could be effective to generate smaller particles.

## Discussion

In this study, an ANNs model was developed to investigate parameters affecting particle size. Our findings showed that flow rate does not appear to be affecting the size extensively. In electrospray process, finding the optimum value for flow rate depends on electrical conductivity of the liquid. If electrical conductivity of the liquid is low, the flow rate will not have a significant effect on particle size ^6^. The data presented in this study indicated that increasing surfactant concentration makes the particle size substantially smaller. In our work, Tween 80, a non-ionic surfactant was used as the stabilizer. Small particles require a sufficient amount of surfactant for stabilization. Our results are consistent with Khani's findings which showed a considerable size reduction following increasing surfactant concentration. Surfactant molecules accumulate on the surface of the particles and prevent the particles from joining together. This prevents the increase of particle size, and with increasing concentrations of Tween 80, a decrease in particle size is expected ^7^. It was also shown that polymer/lipid ratio does not create important changes in the size. The addition of polymer to the solution is necessary in terms of prevention of needle blockage and proper formation of particles. Increasing the amount of polymer leads to an increase in viscosity which could result in production of undesired particles. Adequate polymer to lipid ratio could play a role in preparation of particles ^4^. Contrary to our finding, in a previous report, it has been shown that as the ratio of polymer to lipid increases, particle size increases due to accumulation of additional polymers on the particles ^8^. In our study, due to sufficient lipid content and proper choice of polymer to lipid ratio, no significant effect on size was observed. Lipid concentration was found to have direct effect on the size. Our findings are consistent with a previous study ^9^. The effect of lipid concentration on size is related to extrinsic factors. In the electrospray process, particle size can be reduced by increasing the surface tension. As the lipid concentration increases, the surface tension decreases, resulting in preparation of larger particles ^10^.

## Conclusion

In the present study, SLNs were prepared *via* electrospray process which is a simple one-step procedure. A thorough understanding of input factors was achieved by employing a reliable computational software (ANNs). Among the input factors, concentration of Tween 80 showed to be the most effective parameter; with increasing the concentration of Tween 80, the particle size decreased. The second important parameter was concentration of lipid which had a direct effect on size.
